# Association of EGFR Tyrosine Kinase Inhibitor Treatment With Progression-Free Survival Among Taiwanese Patients With Advanced Lung Adenocarcinoma and EGFR Mutation

**DOI:** 10.3389/fphar.2021.720687

**Published:** 2021-08-09

**Authors:** Po-Yen Chen, Chin-Chou Wang, Chien-Ning Hsu, Chung-Yu Chen

**Affiliations:** ^1^ Master Program in Clinical Pharmacy, School of Pharmacy, Kaohsiung Medical University, Kaohsiung, Taiwan; ^2^ Department of Pharmacy, E-Da Hospital, Kaohsiung, Taiwan; ^3^ Division of Pulmonary and Critical Care Medicine, Department of Internal Medicine, and Department of Occupational Medicine, Kaohsiung Chang Gung Memorial Hospital, Chang Gung University, Kaohsiung, Taiwan; ^4^ Department of Pharmacy, Kaohsiung Chang Gung Memorial Hospital, Kaohsiung, Taiwan; ^5^ Department of Pharmacy, Kaohsiung Medical University Hospital, Kaohsiung, Taiwan; ^6^ Department of Medical Research, Kaohsiung Medical University Hospital, Kaohsiung, Taiwan; ^7^ Center for Big Data Research, Kaohsiung Medical University, Kaohsiung, Taiwan

**Keywords:** gefitinib, erlotinib, afatinib, EGFR mutation, real-world effectiveness

## Abstract

**Background:** There is limited data on the relative survival rate of first-line therapy of gefitinib, erlotinib (first-generation epidermal growth factor receptor-tyrosine kinase inhibitor [EGFR-TKI]), and afatinib (second-generation EGFR-TKI) in patients with EGFR-mutated advanced lung adenocarcinoma in real-world data, especially in the Asian population. This study aimed to compare the relative survival rate of gefitinib, erlotinib, and afatinib in patients with EGFR-mutated advanced lung adenocarcinoma by real-world data in Taiwan.

**Methods:** This retrospective cohort population-based study included untreated adult patients diagnosed with advanced lung adenocarcinoma who were identified in the Taiwan National Health Insurance Research Database between 2014 and 2017. The date of EGFR-mutated advanced lung adenocarcinoma diagnosis was referred as index date. This outcome evaluated overall survival (OS) and time to treatment failure (TTF) between gefitinib, erlotinib, and afatinib. Switching EGFR-TKIs or chemotherapy and new development of brain metastases were proxies of TTF. Estimated relative treatment effects on OS and TTF among EGFR-TKIs were adjusted by inverse probability of treatment weighting (IPTW) in Cox proportional hazards model. Propensity score (PS) matched pair analyses were performed as sensitivity analyses.

**Results:** The study cohort included 3,695 patients initiated with gefitinib, 3,310 with erlotinib, and 3,041 with afatinib. The mean age among the three treatment groups was 70.4 (±11.6), 66.8 (±11.6), and 64.3 (±11.4) years, and the female percentage was 70.4, 58.6, and 57.7%, respectively. Afatinib showed longer median OS than gefitinib (23.9 vs. 21.3 months; adjusted hazard ratio (aHR), 0.87; *p* < 0.001) and erlotinib (23.9 vs. 21.8 months; aHR, 0.87; *p* = 0.001). Consistent results were observed with TTF outcomes. For patients with brain metastases at diagnosis, afatinib showed similar OS with erlotinib (*p* = 0.917) but superior to gefitinib (*p* = 0.028). PS matching had similar results with IPTW adjustment in the study population.

**Conclusion:** Afatinib as first-line therapy had better survival outcomes for EGFR-mutated advanced lung adenocarcinoma than gefitinib and erlotinib in the Taiwan population. Both erlotinib and afatinib demonstrated superior treatment effect in patients with initial brain metastases than gefitinib.

## Introduction

Lung cancer is the major cause of cancer-related death worldwide (Bray et al., 2018; Ministry of Health and Welfare, 2019). Epidermal growth factor receptor (EGFR) is one of the members of ErbB/HER transmembrane receptor family facilitating cellular regulation and proliferation (Chen et al., 2016). EGFR mutation is correlated to neoplasia, and the incidence in the Asian population is higher than that in the Western population (i.e., 57% in Taiwan, 15% in Europe; 22% in North America) (Midha et al., 2015). Of these, EGFR-tyrosine kinase inhibitor (TKI) was recommended by treatment guidelines as first-line treatment for EGFR-mutated advanced non-small cell lung cancer (Hanna et al., 2017; Clinical Practice Gu, 2018; Wu et al., 2019a).

The superior progression-free survival (PFS) of gefitinib, erlotinib (first-generation EGFR-TKI), and afatinib (second-generation EGFR-TKI) compared to platinum-based doublet therapy was confirmed in clinical trials (Maemondo et al., 2010; Mitsudomi et al., 2010; Zhou et al., 2011; Rosell et al., 2012; Sequist et al., 2013; Wu et al., 2014; Wu et al., 2015). Moreover, several trials had been conducted to investigate head-to-head comparison of EGFR-TKIs. The phase III CTONG 0901 trial that enrolled Chinese patients was designed to compare erlotinib and gefitinib, which indicated no significant difference in PFS (13.0 vs. 10.4 months; hazard ratio [HR], 0.81; 95% confidence interval [CI], 0.62–1.05; *p* = 0.108) and overall survival (OS) (22.9 vs. 20.1 months; HR, 0.84; 95% CI, 0.63–1.13, *p* = 0.250) (Yang et al., 2017). Phase IIb Lux-Lung seven trial compared afatinib and gefitinib and revealed longer PFS in the afatinib group (11.0 vs. 10.9 months; *p* = 0.017) but not in OS (27.9 vs. 24.5 months; *p* = 0.258). (Paz-Ares et al., 2017).

Nonetheless, the limited sample size of Asian ethnicity in Lux-Lung seven trial may be underpowered to demonstrate the OS discrepancy with gefitinib and afatinib. Second, patients with unstable brain metastases, cardiovascular abnormalities, or gastrointestinal disorders were excluded in trials that were prevalent in clinical practice. Third, erlotinib demonstrated a higher blood-brain barrier penetration rate than gefitinib and afatinib, which may be beneficial to patients with brain metastases. However, there was still lack of population-based evidence compared to erlotinib and afatinib in these patients (Ahluwalia et al., 2018). Lastly, certain nature of difference between trial design and real-world practice should be considered. To complement knowledge of clinical decision-making, real-world data (RWD) could accommodate a more comprehensive population with longer period to address the choice of preliminary EGFR-TKI treatment.

In Taiwan, gefitinib, erlotinib, and afatinib had been reimbursed in first-line treatment for advanced lung adenocarcinoma harboring EGFR mutation in 2011, 2013, and 2014, respectively. A retrospective study using population-based claims data between 2011 and 2015 showed that afatinib had superior OS than gefitinib (adjusted HR [aHR], 0.82; 95% CI, 0.72–0.93; *p* < 0.001) but not erlotinib (aHR, 0.95; 95% CI, 0.86–1.05, *p* = 0.159) (Hsieh et al., 2019). The study did not address brain metastases at diagnosis, and the limited observation period of afatinib might lack explanation for long-term survival effect. Furthermore, probable selection bias was presented in the imbalance of baseline characteristics.

This study applied the latest RWD to assess the relative survival rate and time to treatment failure (TTF) of gefitinib, erlotinib, and afatinib in first-line therapy for patients with advanced lung adenocarcinoma with EGFR mutation, and propensity score (PS) method was performed to adjust the estimated HR.

## Materials and Methods

### Data Source

The retrospective population-based cohort study was executed using the National Health Insurance Research Database (NHIRD), which covered >99% of the Taiwanese population. The NHIRD included the reimbursement records of inpatient and outpatient visit and emergency admission, which provided patient characteristics, disease diagnoses, and medical treatment. The National Health Insurance Administration (NHIA) collated the claims data yearly to ensure the quality for academic research.

### Study Design and Patient Cohort

Adult patients diagnosed with lung cancer (ICD-9-CM, 162.x; ICD-10-CM, C33, C34) between 2014 and 2017 were identified, and the first prescription date of gefitinib, erlotinib, or afatinib was defined as the index date (ATC code: L01XE02, L01XE03, L01XE13). In Taiwan, first-line use of the three EGFR-TKIs was reimbursement for advanced (stage IIIb, IIIc or IV) lung adenocarcinoma harboring EGFR mutation, and restricted for monotherapy. A pre-audit approval was warranted to confirm the pathology, cell examination report and EGFR testing result. To refill prescription of EGFR-TKI, the chest radiography or computed tomography image must be checked every 4 weeks to evaluate the treatment response. Patients after 2014 were included since three EGFR-TKIs were reimbursed with the same reimbursement criteria. Wash-out period of 1 year was established to ensure patients were treatment naïve and receive gefitinib, erlotinib or afatinib as first-line treatment. The excluded criteria were applied to confirm the study cohort: 1) patients had any cancer diagnoses or previously received other antineoplastic agents (ATC code, L01.x) prior to 1 year of the index date; 2) patients did not have claims information or NHI eligibility by 1 year of index date; 3) patients had unknown sex; 4) patients had coprescription with other antineoplastic drugs on index date. This study was approved by the Institutional Review Board of Ethical Kaohsiung Medical University, Kaohsiung in Taiwan. This study followed the Strengthening the Reporting of Observational Studies in Epidemiology reporting guideline for cohort studies.

### Outcomes

The outcome was divided into OS and TTF. The OS would evaluate survival rate from index date to censor or all-cause death, and the follow-up period was defined from index date to the date of death or end date of follow-up (December 31, 2017). The TTF was the time to treatment failure in the follow-up period, which was used as the proxy of PFS. The follow-up period of TTF was determined from the index date to progressive event or censor; progressive events included switching to or adding another antineoplastic therapy or EGFR-TKI, newly diagnosed brain metastases, and death. If patients did not receive any subsequent antineoplastic therapy after the first EGFR-TKI, the end date of first-line EGFR-TKI treatment was considered a progressive event. Subgroup analysis was conducted according to patients’ brain metastases status at diagnosis.

### Study Variables

Variables including age, sex, year of diagnosis, brain metastases, and comorbidities were retrieved from the database to depict the patient characteristics. In Taiwan, NHIA implemented ICD-10-CM in the medical expenses reporting system in 2015. Due to the different taxonomies on coding algorithms between original ICD-9-CM and ICD-10-CM, we applied enhanced ICD-9-CM in Supplementary Table S1 in Supplement developed from Quan et al.’s study to improve the comparability between the two coding algorithms (Quan et al., 2005). The diagnosis of brain metastases was according to ICD code (ICD-9-CM, 198.3; ICD-10-CM, C79.31). The Charlson’s Comorbidity Index (CCI) was divided into three groups: 0, 1, and d ≧2. All comorbidities were identified 1 year before the index date and confirmed with at least two outpatient records or one inpatient record.

### Statistical Analysis

The descriptive statistics were presented in mean with standard deviation (SD) and median with interquartile range for continuous variables and number and percent for categorical variables. One-way analysis of variance for continuous variables and chi-square test or Fisher’s exact test for categorical variables was used to compare baseline characteristics among three EGFR-TKI arms. Furthermore, standardized mean differences were also used to compare baseline characteristics among the three EGFR-TKI arms before and after PS matching.

The Kaplan-Meier method and log-rank test were applied to estimate the outcome. Cox proportional hazard regression was performed to estimate HR and 95% CI in the univariate and multivariable analyses. Demographic data including age, sex, brain metastases, and CCI score were used for adjustment.

To decrease probable selection bias among EGFR-TKI treatments, the inverse probability of treatment weighting (IPTW) and PS matching were performed for both OS and TTF estimates with variables of age, sex, year of diagnosis, brain metastases, and comorbidities. The weighted approach ensured that patients would not be excluded and mimicked a pseudopopulation to reflect baseline characteristics of the whole population. It remained the representative of the included patients, which was beneficial to generalize the result of the treatment effect (Leslie and Thiebaud, 2007; Brookhart et al., 2013). In PS matching, we initially matched 1:1 with afatinib and gefitinib and then 1:1 with afatinib and erlotinib. Since the two-step PS matching would certainly decrease the sample size, the matched method was conducted for sensitivity analysis.

Two-sided *p*-value with <0.05 was considered statistically significant. SMD >0.1 was considered as imbalance in the two groups. SAS 4.0 (SAS Institute, Cary, NC, United States) was used to conduct the data analyses.

## Results

### Patient Characteristics

Overall, 93,137 patients with lung cancer were identified from January 1, 2014 and December 31, 2017. After excluding patients aged <20 years, 17,522 patients received either gefitinib, erlotinib, or afatinib following the first lung cancer diagnosis. Next, patients with prior antineoplastic therapy or pre-existing cancer (*n* = 7,162), lack of prior claims information or NHI eligibility (*n* = 213), unknown sex (*n* = 22), and coprescription with other antineoplastic agents (*n* = 79) were excluded. A total of 10,046 patients with EGFR-mutant advanced lung adenocarcinoma initiated first-line EGFR-TKI therapy with 3,695 patients who received gefitinib, 3,310 who received erlotinib and 3,041 who received afatinib (Supplementary Figure S1 in Supplement).

Table 1 shows the baseline characteristics of the three groups. The SMD was revealed in age, sex, year of diagnosis, brain metastases status, and CCI score. The age of the afatinib group (mean ± SD, 64.3 ± 11.4) was lower than those of the gefitinib (70.4 ± 11.6) and erlotinib groups (66.8 ± 11.6), whereas the percentages of elderly patients were 47.9, 68.9, and 57.3%, respectively. More female patients were prescribed with gefitinib (70.4%) than with erlotinib (58.6%) and afatinib (57.7%). Patients with brain metastases at baseline tended to receive erlotinib as first-line treatment compared to gefitinib and afatinib. The proportions of severe comorbidity (CCI score ≥2) in the gefitinib, erlotinib, and afatinib groups were 74.6, 79.2, and 70.8%, respectively. Patient characteristics after IPTW and PS matching are shown in Supplementary Tables S2A,B in Supplement.

**TABLE 1:** Baseline characteristics.

	N	%	Gefitinib		Erlotinib		Afatinib		*p* value	Gefitinib vs. Erlotinib	Gefitinib vs. Afatinib	Erlotinib vs. Afatinib
			N	%	N	%	N	%		SMD	SMD	SMD
**Total**	10,046		3,695		3,310		3,041					
**Age**												
Mean (±SD)	67.4	11.8	70.4	11.6	66.8	11.6	64.3	11.4	<0.001	0.31	0.53	0.22
20–64	4,146	41.27	1,151	31.15	1,412	42.66	1,583	52.06	<0.001	0.24	0.43	0.19
≧65	5,900	58.73	2,544	68.85	1,898	57.34	1,458	47.94				
**Gender**												
Male	3,753	37.36	1,094	29.61	1,372	41.45	1,287	42.32	<0.001	0.25	0.27	0.02
Female	6,293	62.64	2,601	70.39	1,938	58.55	1,754	57.68				
**Diagnosed year**												
2014	2,360	23.49	1,164	31.50	880	26.59	316	10.39	<0.001	0.11	0.54	0.43
2015	2,476	24.65	998	27.01	752	22.72	726	23.87		0.10	0.07	0.03
2016	2,496	24.85	782	21.16	798	24.11	916	30.12		0.07	0.21	0.14
2017	2,714	27.02	751	20.32	880	26.59	1,083	35.61		0.15	0.35	0.20
**Comorbidity**												
Myocardial infarction	94	0.94	33	0.89	36	1.09	25	0.82	0.517	0.02	0.01	0.03
Congestive heart failure	579	5.76	280	7.58	157	4.74	142	4.67	<0.001	0.12	0.12	0.00
Peripheral vascular disease	136	1.35	50	1.35	52	1.57	34	1.12	0.296	0.02	0.02	0.04
Cerebrovascular disease	1,053	10.48	457	12.37	356	10.76	240	7.89	<0.001	0.05	0.15	0.10
Dementia	329	3.27	157	4.25	108	3.26	64	2.10	<0.001	0.05	0.12	0.07
Chronic pulmonary disease	2,774	27.61	998	27.01	928	28.04	848	27.89	0.582	0.02	0.02	0.00
Rheumatic disease	120	1.19	48	1.30	38	1.15	34	1.12	0.758	0.01	0.02	0.00
Peptic ulcer disease	1462	14.55	596	16.13	457	13.81	409	13.45	0.003	0.07	0.08	0.01
Mild liver disease	642	6.39	210	5.68	210	6.34	222	7.30	0.026	0.03	0.07	0.04
Diabetes without chronic complication	2070	20.61	824	22.30	694	20.97	552	18.15	<0.001	0.03	0.10	0.07
Diabetes with chronic complication	538	5.36	223	6.04	187	5.65	128	4.21	0.003	0.02	0.08	0.07
Hemiplegia/paraplegia	101	1.01	42	1.14	38	1.15	21	0.69	0.114	0.00	0.05	0.05
Renal disease	593	5.90	278	7.52	177	5.35	138	4.54	<0.001	0.09	0.13	0.04
Moderate-severe liver disease	7	0.07	<5		<5		<5			0.01	0.03	0.04
Metastatic solid tumor	6,365	63.36	2,255	61.03	2,279	68.85	1831	60.21	<0.001	0.16	0.02	0.18
AIDS/HIV	<5		<5		<5		<5			0.01	0.03	0.02
**CCI score**												
0	1271	12.65	465	12.58	345	10.42	461	15.16	<0.001	0.07	0.07	0.14
1	1247	12.41	475	12.86	344	10.39	428	14.07		0.08	0.04	0.11
≧2	7,528	74.94	2,755	74.56	2,621	79.18	2,152	70.77		0.11	0.09	0.20
**Brain metastases**												
Yes	1995	19.86	490	13.26	959	28.97	546	17.95	<0.001	0.39	0.13	0.26
No	8051	80.14	3205	86.74	2351	71.03	2495	82.05				

Abbreviations: SD: standard deviation; SMD: standard mean difference; AIDS: acquired immunodeficiency syndrome; HIV: Human Immunodeficiency Virus; CCI: Charlson Comorbidity Index.

### Overall Survival

The median follow-up durations in the gefitinib, erlotinib, and afatinib groups were 16.4, 15.1, and 13.8 months. Kaplan–Meier estimates and log-rank test showed that afatinib was associated with longer OS compared with gefitinib (*p* < 0.001) and erlotinib (*p* < 0.001) and the median OS was 25.5, 20.9, and 21.4 months, respectively (Supplementary Figure S2A in Supplement). After IPTW, the median OS of the afatinib group was 23.9 vs. 21.3 months in the gefitinib group (*p* < 0.001) and 21.8 months in the erlotinib group (*p* = 0.001) (Supplementary Figure S2B). The 1-year survival rates of gefitinib, erlotinib, and afatinib were 70.0, 71.6, and 74.4%, and the 2-years survival rates were 44.7, 47.6, and 50.3%, respectively.

Multivariable analyses demonstrated that afatinib had lower mortality rate than gefitinib (aHR = 0.864; 95% CI, 0.804 to 0.928; *p* < 0.001) and erlotinib (aHR = 0.879; 95% CI, 0.817 to 0.946; *p* < 0.001) (Table 2). Baseline factors related to hazard of death included old age, male sex, severe comorbidity (CCI score≧2), and baseline brain metastases.

**TABLE 2:** Cox proportional hazard model for overall survival after IPTW.

	Univariate analysis			Multivariable analysis		
Variables	Crude HR	95% CI	*p* value	Adjusted HR	95% CI	*p* value
**EGFR-TKI**						
Erlotinib vs. Gefitinib	0.996	0.930–1.066	0.902	0.983	0.918–1.052	0.617
Afatinib vs. Gefitinib	0.868	0.808–0.932	<0.001	0.864	0.804–0.928	<0.001
Afatinib vs. Erlotinib	0.872	0.810–0.938	<0.001	0.879	0.817–0.946	<0.001
**Age**						
20–64	1.000			1.000		
≧65	1.407	1.325–1.494	<0.001	2.553	2.202–2.961	<0.001
**Gender**						
Male	1.000			1.000		
Female	0.795	0.750–0.843	<0.001	0.697	0.614–0.791	<0.001
**CCI score**						
0	1.000			1.000		
1	1.165	1.021–1.331	0.024	1.085	0.950–1.240	0.229
≧2	1.813	1.640–2.005	<0.001	1.605	1.448–1.779	<0.001
**Brain metastases**						
No	1.000			1.000		
Yes	1.434	1.340–1.534	<0.001	1.325	1.234–1.422	<0.001

Abbreviations: HR: hazard ratio; CI: confidence interval; EGFR-TKI: epidermal growth factor receptor-tyrosine kinase inhibitor; CCI: Charlson Comorbidity Index.

### Time to Treatment Failure

The median follow-up period in the gefitinib, erlotinib, and afatinib groups were 9.5, 9.9, and 10.1 months, respectively. Afatinib showed superior TTF against gefitinib (*p* < 0.001) and erlotinib (*p* < 0.001), and the median TTFs were 11.7, 9.7, and 9.7 months, respectively (Supplementary Figure S3A in Supplement). After IPTW, the median TTF of afatinib was 11.5 vs. 9.5 months in the gefitinib group (*p* < 0.001) and 9.7 months in the erlotinib group (*p* < 0.001) (Supplementary Figure S3B). The 1-year non-failure rates in the gefitinib, erlotinib, and afatinib were 40.1, 43.2, and 49.6%, and the 2-years estimated rates were 14.3, 16.1, and 21.3%, respectively.

Table 3 shows the multivariable analysis that revealed similar results with OS estimates. Patients in the afatinib group were less likely to have treatment failure with aHR of 0.817 (95% CI, 0.772 to 0.864, *p* < 0.001) vs. gefitinib and 0.851 (95% CI, 0.803 to 0.902, *p* < 0.001) vs. erlotinib. The factors associated with treatment failure were consistent with the results of OS.

**TABLE 3:** Cox proportional hazard model for time to treatment failure after IPTW.

	Univariate analysis			Multivariable analysis		
Variables	Crude HR	95% CI	*p* value	Adjusted HR	95% CI	*p* value
**EGFR-TKI**						
Erlotinib vs. Gefitinib	0.996	0.915–1.020	0.216	0.959	0.908–1.013	0.137
Afatinib vs. Gefitinib	0.820	0.775–0.868	<0.001	0.817	0.772–0.864	<0.001
Afatinib vs. Erlotinib	0.849	0.801–0.900	<0.001	0.851	0.803–0.902	<0.001
**Age**						
20–64	1.000			1.000		
≧65	1.030	0.983–1.079	0.214	1.304	1.192–1.426	<0.001
**Gender**						
Male	1.000			1.000		
Female	0.873	0.833–0.915	<0.001	0.807	0.741–0.879	<0.001
**CCI score**						
0	1.000			1.000		
1	1.078	0.979–1.186	0.127	1.058	0.960–1.165	0.257
≧2	1.361	1.266–1.464	<0.001	1.284	1.191–1.383	<0.001
**Brain metastases**						
No	1.000			1.000		
Yes	1.264	1.196–1.337	<0.001	1.137	1.024–1.261	0.016

Abbreviations: SD: standard deviation; EGFR-TKI: epidermal growth factor receptor-tyrosine kinase inhibitor; CCI: Charlson Comorbidity Index.

### Subgroup Analysis

The subgroup analysis focused on patients with or without brain metastases at diagnosis. In brain metastases, the crude OS in the gefitinib, erlotinib, and afatinib groups were 15.3, 18.7, and 19.8 months (Supplementary Figure S2C). IPTW estimates were 15.7, 18.7, and 18.3 months, respectively (Supplementary Figure S2D). The crude TTF in the three study groups were 8.5, 9.5, and 11.7 months (Supplementary Figure S3C); IPTW estimates were 8.4, 9.4, and 9.2 months, respectively (Supplementary Figure S3D). OS and TTF were similar between erlotinib and afatinib, and both treatments were superior to the gefitinib group.

In the absence of brain metastases (Supplementary Figures S4, S5 in Supplement), the crude OS in the gefitinib, erlotinib, and afatinib groups were 21.9, 23.0, and 28.4 months; IPTW estimates were 22.6, 23.1, and 25.6 months, respectively. The crude TTF in the three study groups were 10.1, 9.8, and 12.0 months; IPTW estimates were 9.9, 9.9, and 11.9 months, respectively. The afatinib group presented better outcomes compared with groups that received first-generation EGFR-TKI, and there was no statistical difference between the gefitinib and erlotinib groups.

### Sensitivity Analysis

PS matching method was conducted for sensitivity analysis. Supplementary Figure S6 in Supplement showed PS matching-adjusted OS was 22.1 months with gefitinib, 22.6 months with erlotinib, and 24.9 months with afatinib. Afatinib had better survival compared with gefitinib (*p* = 0.007) and erlotinib (*p* = 0.004). Multivariable analysis favored the afatinib group compared to the gefitinib (aHR = 0.819, 95% CI, 0.735 to 0.912, *p* < 0.001) and erlotinib groups (aHR = 0.870, 95% CI, 0.781 to 0.968, *p* = 0.011) (Supplementary Table S3 in Supplement).

The PS matching-adjusted TTF in the three groups were 9.6, 9.9, and 11.7 months, respectively (Supplementary Figure S7 in Supplement). The TTF was significantly longer for patients receiving afatinib (<0.001 vs. those receiving gefitinib and erlotinib). Multivariable analysis revealed that afatinib was associated with aHR of 0.869 (95% CI, 0.764 to 0.988; *p* = 0.032) vs. gefitinib and 0.892 (95% CI, 0.784 to 1.015; *p* = 0.083) vs. erlotinib (Supplementary Table S4 in Supplement).

## Discussion

This study was conducted with the latest population-based RWD to evaluate relative survival rate and TTF of first-line treatment for EGFR-mutated advanced lung adenocarcinoma in Taiwan. Median OS and TTF were comparable with the results in clinical trials (Park et al., 2016; Yang et al., 2017). The slightly prolonged survival outcomes in the trial setting may result from the inclusion of patients who were younger (CTONG 0901, 58.5 years; Lux-Lung 7, 63 years; RWD, 67 years) and had less baseline brain metastases (CTONG 0901. 18.4%; Lux-Lung 7, 15.7%; RWD, 19.8%) and fewer comorbidities than those of RWD. The present study revealed that patients who preliminarily received afatinib had superior OS and TTF compared with those who received gefitinib and erlotinib, while a similar treatment effect was shown in the first-generation EGFR-TKIs. In OS estimates, Lux-Lung seven trial demonstrated no significant difference between the afatinib and gefitinib groups, but a discrepancy of 3.4 months was observed. The treatment benefit of afatinib in the Asian population was confirmed with sufficient sample size in the real-world situation.

The rigorous reimbursement criteria and regular treatment evaluation ensure consecutive EGFR-TKI treatment toward target patients in Taiwan. Given the real-world setting, the primary choice of EGFR-TKI is subject to the patient characteristics at diagnosis, and the baseline distribution in our study is in concordance with clinical experience. Patients who received afatinib were younger and had relatively mild comorbidity, which was supposed to prevent adverse events. Gefitinib tended to be prescribed for women, and erlotinib was commonly used for patients with initial brain metastases. The prescription patterns were in line with those of previous studies (Park et al., 2016; Hsieh et al., 2019; Kim et al., 2019; Ito et al., 2020). To facilitate the relative effectiveness assessment, two well-established PS methods were performed to adjust the survival. The similar hazard of treatment failure and mortality verified the treatment effect among the three EGFR-TKI treatments.

For patients initially diagnosed with EGFR-mutant advanced lung adenocarcinoma, concurrent brain metastasis was associated with severe morbidity, poor survival outcomes, and quality of life (Economopoulou and Mountzios, 2016). Previous direct comparison of EGFR-TKIs for these patients was investigated in Lux-Lung seven trial, but the sample size between the gefitinib (*n* = 24) and afatinib (*n* = 26) groups was limited. In the present study, brain metastasis was prevalent with up to 20% in our cohort, and results exhibited that both erlotinib and afatinib had better OS and TTF compared to gefitinib. As previous research, blood-brain barrier penetration rate of afatinib was lower than erlotinib (Ahluwalia et al., 2018). However, other research showed afatinib permeability rate was enough to inhibit tumor cell and present clinical improvement (Hoffknecht et al., 2015). Moreover, afatinib concentration in CSF was correlated to treatment dosage, so the adverse event tolerability and effective treatment dose should be pondered in clinical practice. Based on the claims data of large sample size, the result provided real-world evidence to address the front-line choice for the subgroup patients.

The third-generation EGFR-TKI osimertinib demonstrated superior treatment benefit compared to gefitinib/erlotinib in the first-line therapy (Soria et al., 2018). However, the drug accessibility was scant because of the high acquisition price and unfavorable cost-effectiveness results worldwide (Aguiar et al., 2018; Ezeife et al., 2018; Wu et al., 2019b; Cai et al., 2019). In Taiwan, osimertinib was covered by the national health insurance program in 2020 and conditionally reimbursed for stage IV non-brain metastatic lung adenocarcinoma as first-line treatment, and patients progressed after first-line gefitinib, erlotinib, or afatinib therapy harboring T790M mutation. Untreated patients who had initial brain metastases could only receive first- or second-generation EGFR-TKI, and the present study supported erlotinib and afatinib to be the best treatment option. Furthermore, a previous study indicated that progressive events in patients receiving first- or second-generation EGFR-TKI mostly originated from T790M mutation (Oxnard et al., 2011; Yu et al., 2013). The secondary EGFR mutation also accounted for most frequent mechanisms of EGFR-TKI resistance in Taiwan (Huang et al., 2018; Lin et al., 2019). Hence, in patients with baseline brain metastases, the front-line treatment option of erlotinib or afatinib would not influence hereafter sequential therapy. Other considerations were treatment-related adverse events. Skin reactions, general disorders, and administration site conditions were commonly associated with EGFR-TKIs in trials and real-world settings; generally, patients are considered to tolerate well every EGFR-TKI (Huang et al., 2020).

## Limitation

Several limitations should be noted in the study. First, the nature of non-randomized study design led to selection bias among treatment groups. Otherwise, the limitation of NHIRD leads to lack of smoking status, severity of cancer stage, lifestyle, examination results, performance status, and laboratory data. To our best effort, IPTW and PS matching was applied to adjust well-known prognostic factors in lung cancer treatment, and results in the Cox regression model were robust. Second, TTF used as proxy of PFS could exaggerate the time to progression, although patients with initial response or slow progression may continue EGFR-TKI with local therapy. The TTF endpoint may be more practical to reflect the contribution of EGFR-TKI treatment. Third, the compassionate use of osimertinib was approved in 2016. Approximately 1,000 patients received osimertinib as subsequent therapy, which may compromise relative outcome. Nonetheless, treatment failure rate was higher in first-line gefitinib and erlotinib, which could crossover to use osimertinib, and the results indirectly strengthened treatment benefit of afatinib. Fourth, despite the latest claims data, the follow-up period may not be comprehensive enough to reflect the lifelong duration. Lastly, NHIRD did not provide mutation type information (i.e., L858R or T790M mutation) in our study period. A meta-analysis showed patients with exon 19 deletion had lower risk of progression than L858R mutation (Lee et al., 2015), but only Lux-Lung seven trial conducted the head-to-head comparison of EGFR-TKIs in both mutation types (Paz-Ares et al., 2017). The divergence of OS curves was observed in patients harboring exon 19 mutation, yet the sample size might be underpowered. Future studies with detailed types of mutation and sufficient sample size are warranted to provide a further prospect for direct comparison of EGFR-TKIs.

## Conclusion

This RWD with large population suggested that afatinib had longer survival rate and TTF than gefitinib and erlotinib in the Asian population. The treatment preference was shown in gefitinib over female patients; erlotinib over patients with brain metastases; and afatinib over younger patients. In patients with brain metastases, both erlotinib and afatinib contribute to longer OS compared with gefitinib. IPTW and PS matching method enhanced the robustness of estimated OS and progression outcomes. Further research could focus on the sequential therapy in patients with different EGFR mutation types.

## Data Availability

The datasets presented in this article are not readily available because legal restrictions imposed by the government of Taiwan in relation to the “Personal Information Protection Act”. Corresponding author (C-YC) had full access to all the data in the study and takes responsibility for the integrity of the data and the accuracy of the data analysis. Data are available from the National Health Insurance Research Database (NHIRD) published by the Bureau of National Health Insurance (BNHI) of the Ministry of Health and Welfare. Requests to access the datasets should be directed to jk2975525@hotmail.com.
