# Supplementary material for: Association of EGFR Tyrosine Kinase Inhibitor Treatment With Progression-Free Survival Among Taiwanese Patients With Advanced Lung Adenocarcinoma and EGFR Mutation
Source: Front Pharmacol. 2021 Aug 9;12:720687. doi: 10.3389/fphar.2021.720687 (PMC8382571; doi:10.3389/fphar.2021.720687)
Supplement: Supplementary file 1 [file DataSheet2.pdf]

Association of EGFR tyrosine kinase inhibitor treatment with progression-free survival among Taiwanese patients with advanced lung adenocarcinoma and EGFR mutation

Supplemental file

eFigure 1 Inclusions and exclusions of study cohort
eFigure 2 Overall survival. Unadjusted base-case analyses (A), IPTW adjusted base-case analyses (B), unadjusted brain metastases subgroup analyses (C), IPTW adjusted brain metastases subgroup analyses (D).
eFigure 3 Time to treatment failure. Unadjusted base-case analyses (A), IPTW adjusted base-case analyses (B), unadjusted brain metastases subgroup analyses (C), IPTW adjusted brain metastases subgroup analyses (D).
eFigure 4 Overall survival in unadjusted non-brain metastases subgroup analyses (A), IPTW adjusted non-brain metastases subgroup analyses (B)
eFigure 5 Time to treatment failure in unadjusted non-brain metastases subgroup analyses (A), IPTW adjusted non-brain metastases subgroup analyses (B)
eFigure 6 Overall survival after propensity score matching in the base-case analyses
eFigure 7 Time to treatment failure after propensity score matching in the base-case analyses

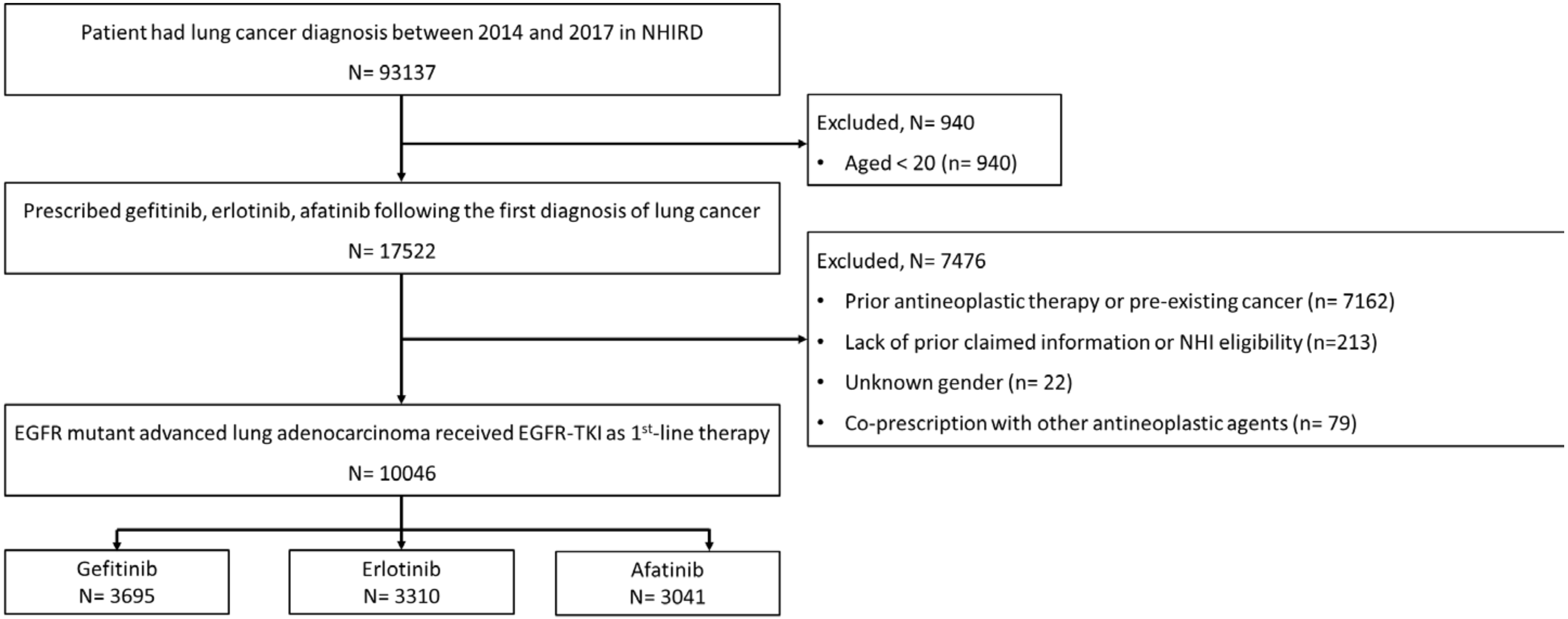

eFigure 1 Inclusions and exclusions of study cohort

NHIRD: National Health Insurance Research Database; EGFR: epidermal growth factor receptor; TKI: tyrosine kinase inhibitor

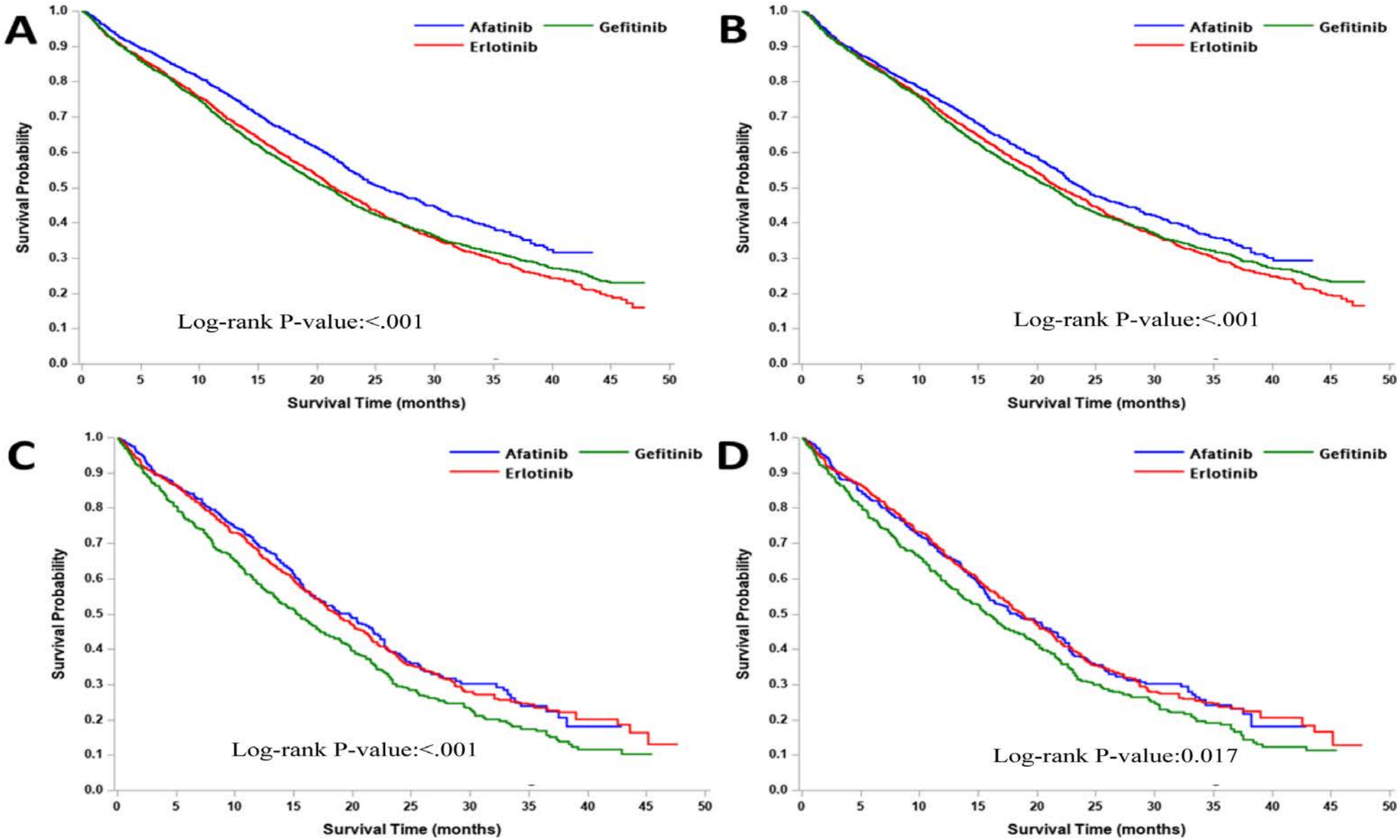

eFigure 2 Overall survival. Unadjusted base-case analyses (A), IPTW adjusted base-case analyses (B), unadjusted brain metastases subgroup analyses (C), IPTW adjusted brain metastases subgroup analyses (D).

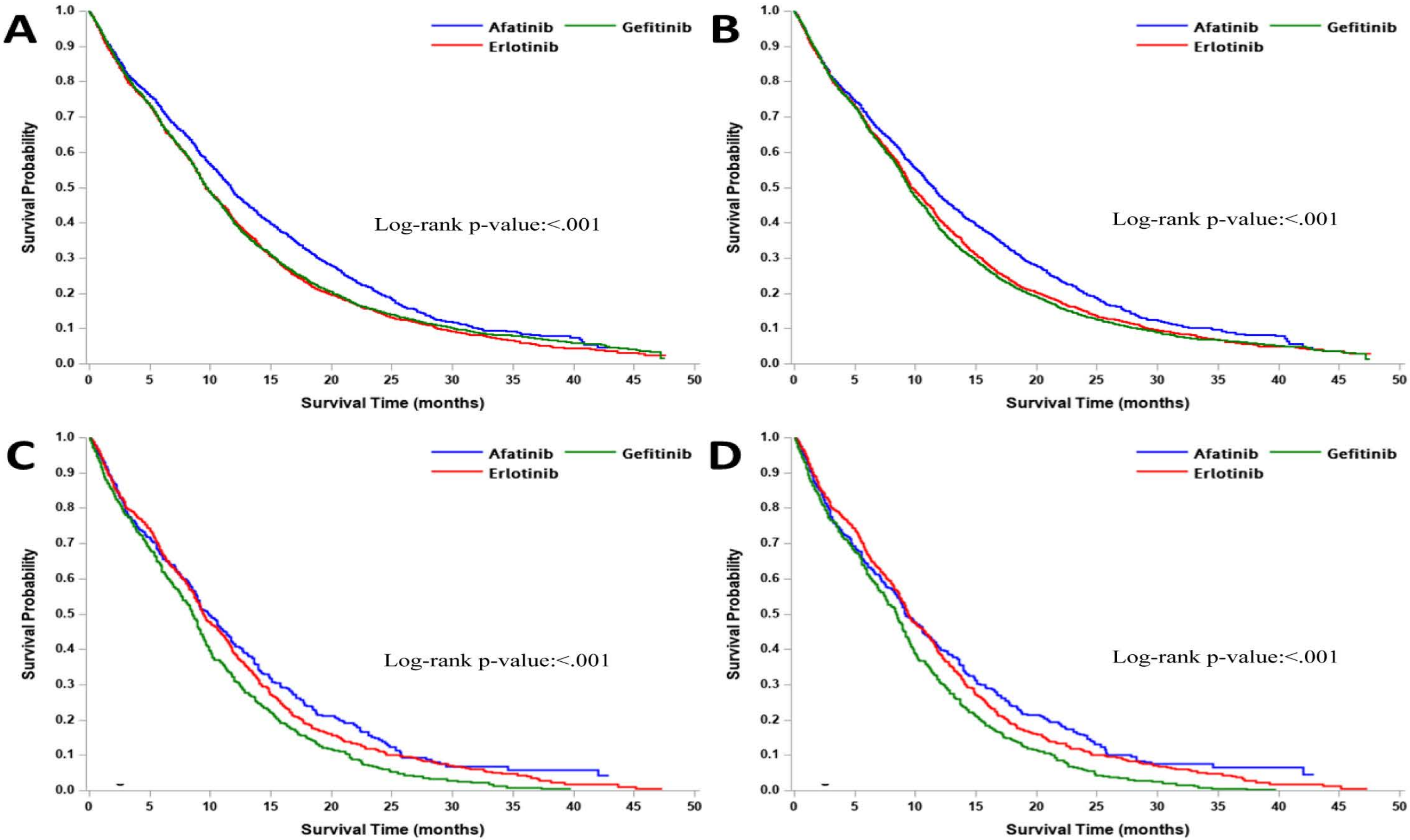

eFigure 3 Time to treatment failure. Unadjusted base-case analyses (A), IPTW adjusted base-case analyses (B), unadjusted brain metastases subgroup analyses (C), IPTW adjusted brain metastases subgroup analyses (D).

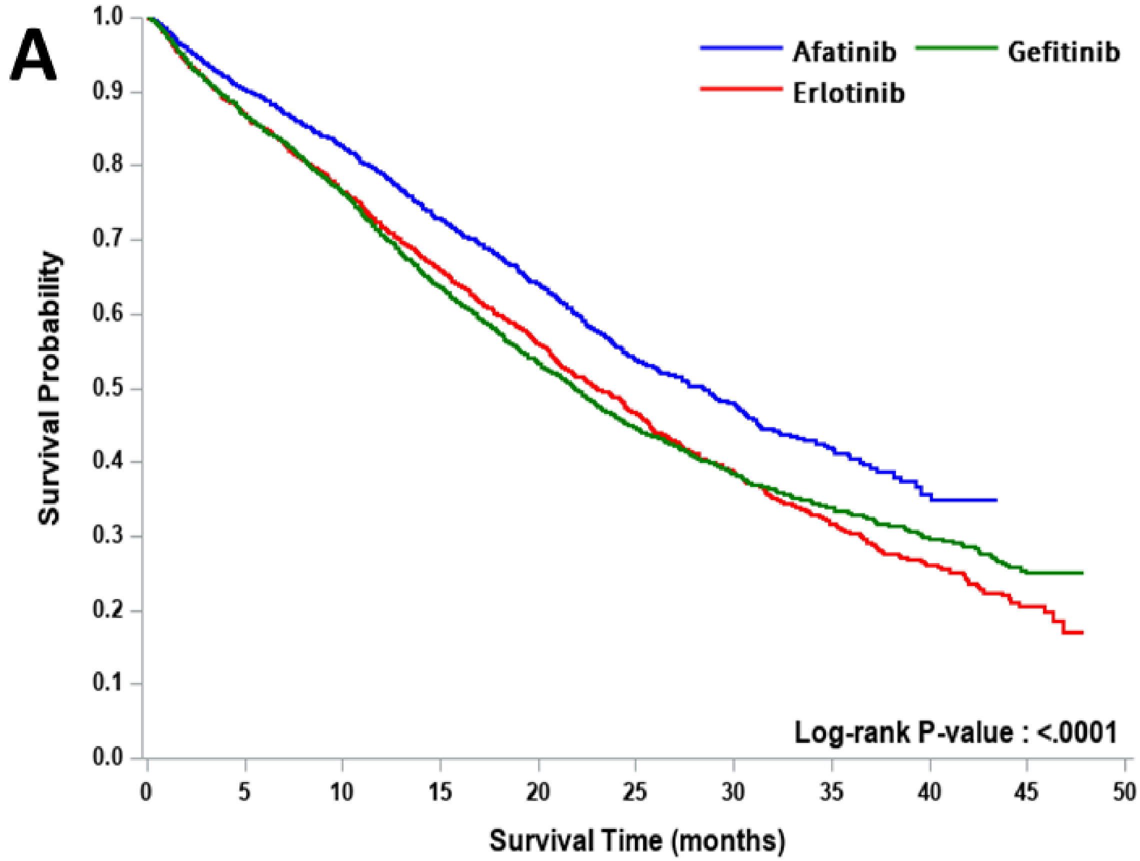

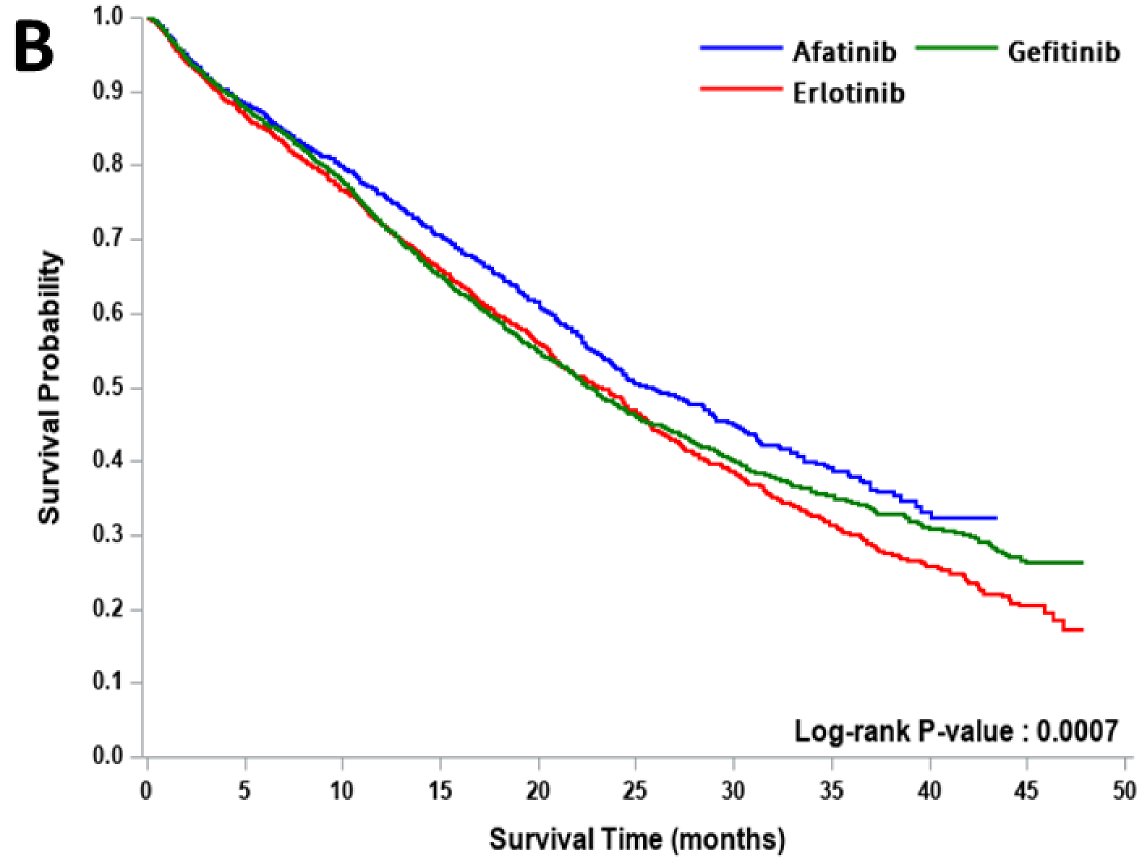

eFigure 4 Overall survival in unadjusted non-brain metastases subgroup analyses (A), IPTW adjusted non-brain metastases subgroup analyses (B)

IPTW: inverse probability of treatment weighting

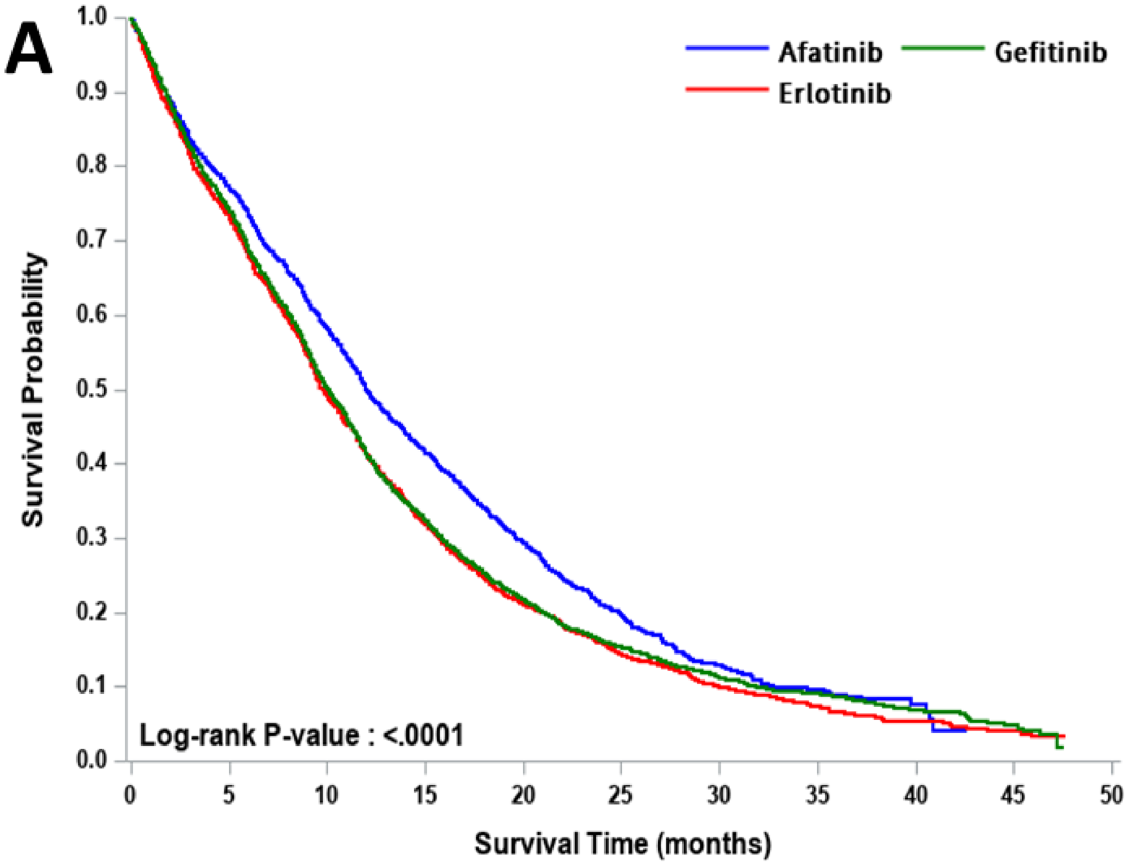

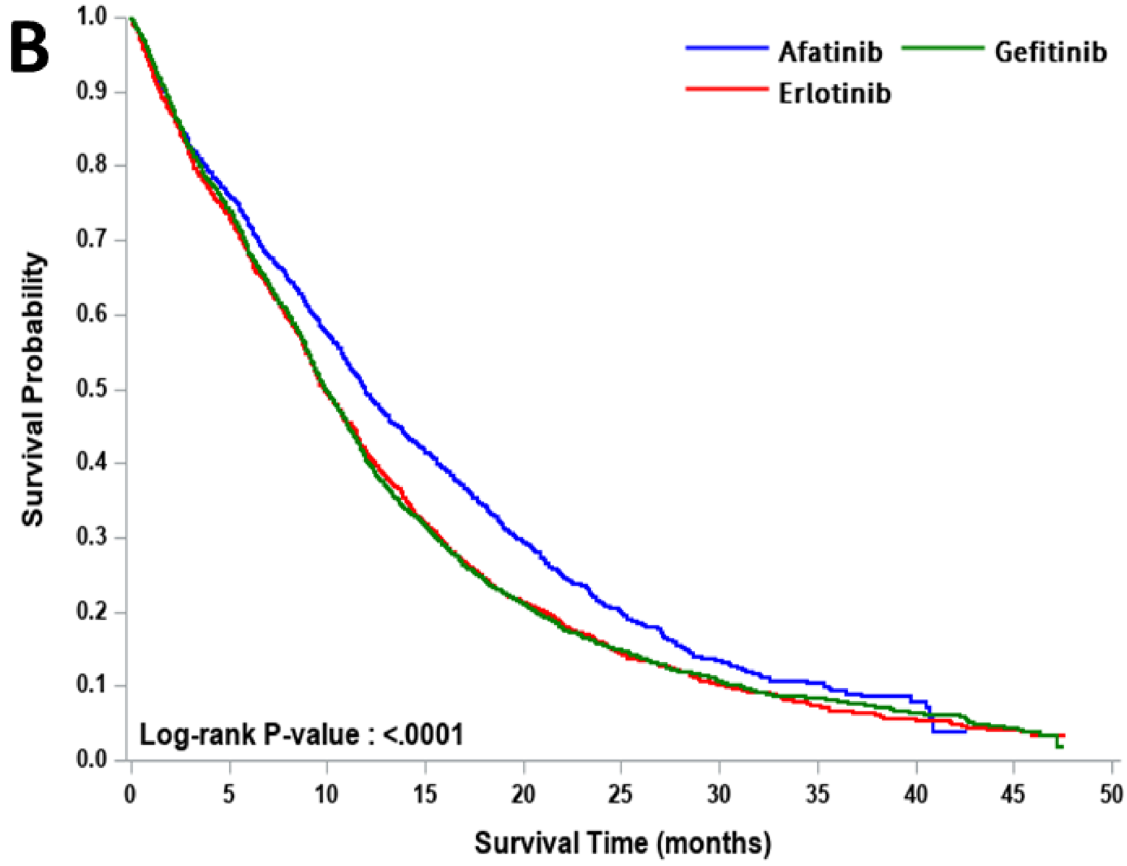

eFigure 5 Time to treatment failure in unadjusted non-brain metastases subgroup analyses (A), IPTW adjusted non-brain metastases subgroup analyses (B)

IPTW: inverse probability of treatment weighting

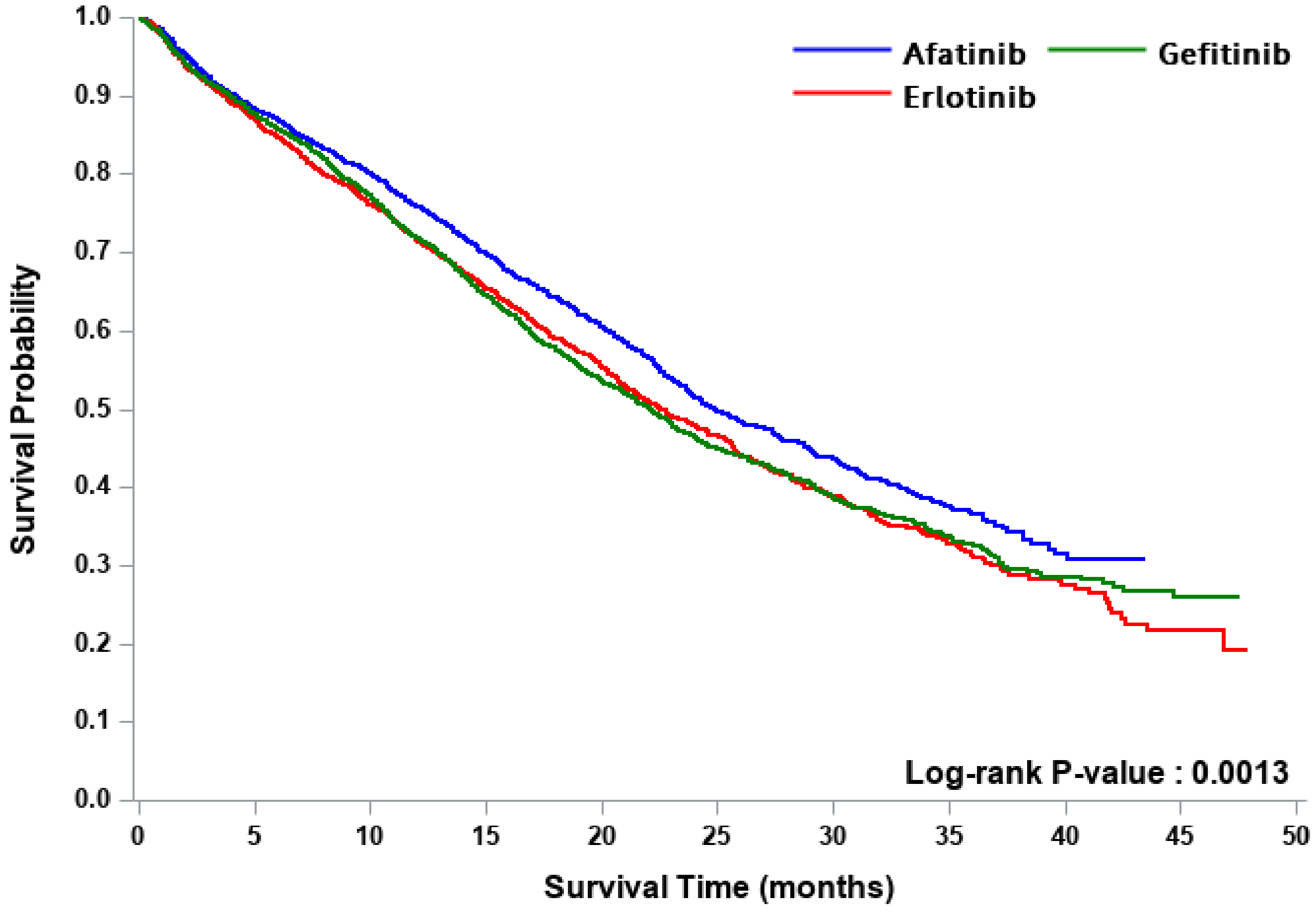

eFigure 6 Overall survival after propensity score matching in the base-case cohort

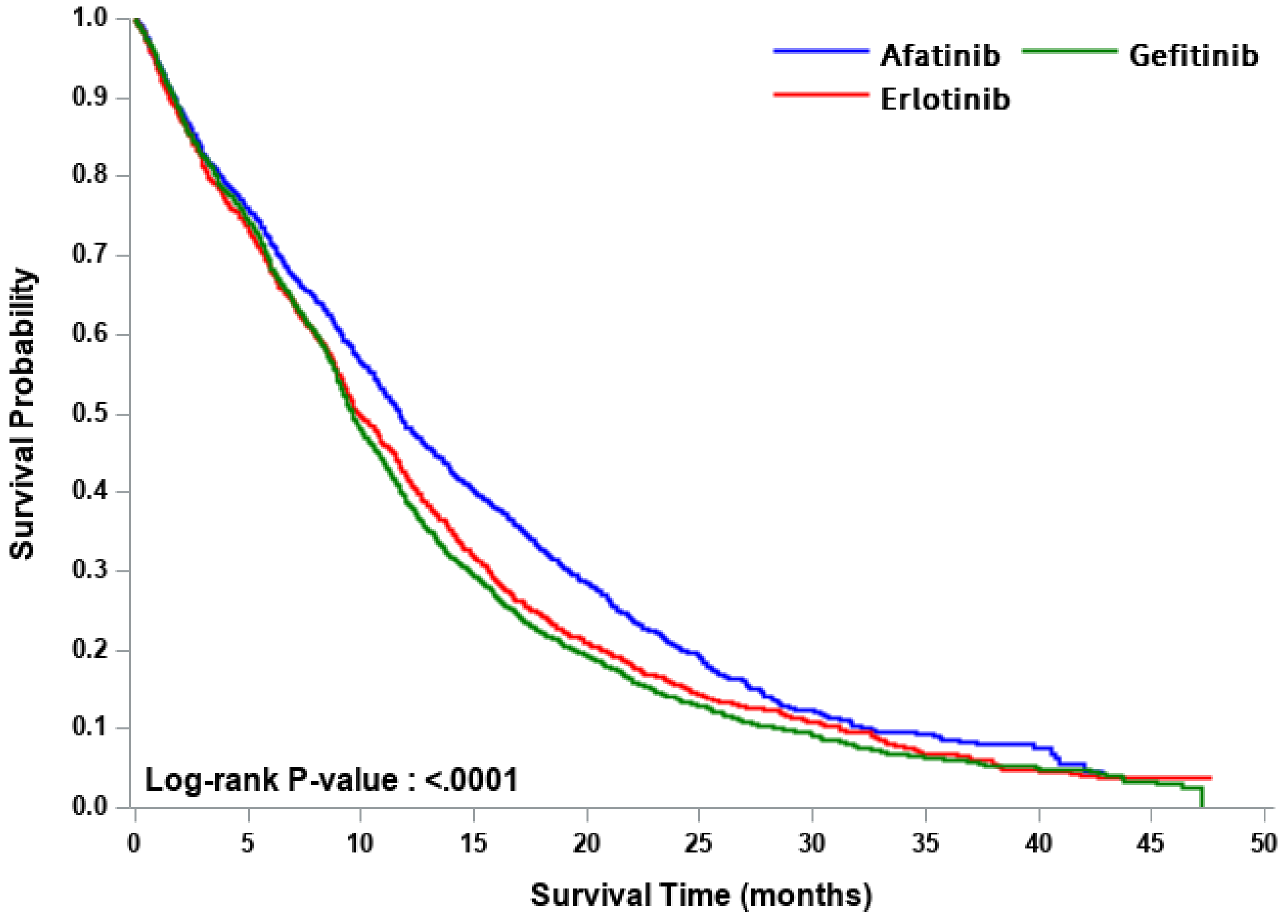

eFigure 7 Time to treatment failure after propensity score matching in the base-case cohort
