# Supplementary material for: Association of EGFR Tyrosine Kinase Inhibitor Treatment With Progression-Free Survival Among Taiwanese Patients With Advanced Lung Adenocarcinoma and EGFR Mutation
Source: Front Pharmacol. 2021 Aug 9;12:720687. doi: 10.3389/fphar.2021.720687 (PMC8382571; doi:10.3389/fphar.2021.720687)
Supplement: Supplementary file 2 [file DataSheet1.docx]

**Association of EGFR tyrosine kinase inhibitor treatment with progression-free survival among Taiwanese patients with advanced lung adenocarcinoma and EGFR mutation**

eTable 1 Coding algorithms for Charlson comorbidities

eTable 2a Baseline characteristics after IPTW

eTable 2b Baseline characteristics after IPTW and propensity score matching

eTable 3 Cox proportional hazard model for overall survival after propensity score matching

eTable 4 Cox proportional hazard model for time to treatment failure after propensity score matching

eTable 1 Coding algorithms for Charlson comorbidities

|  | ICD-9-CM | ICD-10-CM |
| --- | --- | --- |
| Myocardial infarction | 410.x, 412.x | I21.x, I22.x, I25.2 |
| Congestive heart failure | 398.91, 402.01, 402.11, 402.91, 404.01, 404.03, 404.11, 404.13, 404.91, 404.93, 425.4-425.9, 428.x | I09.9,I11.0, I13.0, I13.2, I25.5, I42.0, I42.5-I42.9, I43.x, I50.x, P29.0 |
| Peripheral vascular disease | 093.0, 437.3, 440.x, 441.x, 443.1-443.9, 447.1, 557.1, 557.9, V43.4 | I70.x, I71.x, I73.1, I73.8, I73.9, I77.1, I79.0, I79.2, K55.1, K55.8, K55.9, Z95.8, Z95.9 |
| Cerebrovascular disease | 362.34, 430.x-438.x | G45.x, G46.x, H34.0, I60.x-I69.x |
| Dementia | 290.x, 294.1, 331.2 | F00.x-F03.x, F05.1, G30.x, G31.1 |
| Chronic pulmonary disease | 416.8, 416.9, 490.x-505.x, 506.4, 508.1, 508.8 | I27.8, I27.9, J40.x-J47.x, J60.x-J67.x, J68.4, J70.1, J70.3 |
| Rheumatic disease | 446.5, 710.0-710.4, 714.0-714.2, 714.8, 725.x | M05.x, M06.x, M31.5, M32.x-M34.x, M35.1, M35.3, M36.0 |
| Peptic ulcer disease | 531.x-534.x | K25.x-K28.x |
| Mild liver disease | 070.22, 070.23, 070.32, 070.33, 070.44, 070.54, 070.6, 070.9, 570.x, 571.x, 573.3, 573.4, 573.8, 573.9, V42.7 | B18.x, K70.0-K70.3, K70.9, K71.3-K71.5, K71.7, K73.x, K74.x, K76.0, K76.2-K76.4, K76.8, K76.9, Z94.4 |
| Diabetes without chronic complication | 250.0-250.3, 250.8, 250.9 | E10.0, E10.l, E10.6, E10.8, E10.9, E11.0, E11.1, E11.6, E11.8, E11.9, E12.0, E12.1, E12.6, E12.8, E12.9, E13.0, E13.1, E13.6, E13.8, E13.9, E14.0, E14.1, E14.6, E14.8, E14.9 |
| Diabetes with chronic complication | 50.4-250.7 | E10.2-E10.5, E10.7, E11.2-E11.5, E11.7, E12.2-E12.5, E12.7, E13.2-E13.5, E13.7, E14.2-E14.5, E14.7 |
| Hemiplegia or paraplegia | 334.1, 342.x, 343.x, 344.0-344.6, 344.9 | G04.1, G11.4, G80.1, G80.2, G81.x, G82.x, G83.0-G83.4, G83.9 |

eTable 1 Coding algorithms for Charlson comorbidities (continue)

| Renal disease | 403.01, 403.11, 403.91, 404.02, 404.03, 404.12, 404.13, 404.92, 404.93, 582.x, 583.0-583.7, 585.x, 586.x, 588.0, V42.0, V45.1, V56.x | I12.0, I13.1, N03.2-N03.7, N05.2-N05.7, N18.x, N19.x, N25.0, Z49.0-Z49.2, Z94.0, Z99.2 |
| --- | --- | --- |
| Moderate or severe liver disease | 140.x-172.x, 174.x-195.8,  200.x-208.x, 238.6 | C00.x-C26.x, C30.x-C34.x, C37.x-C41.x, C43.x, C45.x-C58.x, C60.x-C76.x, C81.x-C85.x, C88.x, C90.x-C97.x |
| Metastatic solid tumor | 456.0-456.2, 572.2-572.8 | I85.0, I85.9, I86.4, I98.2, K70.4, K71.1, K72.1, K72.9, K76.5, K76.6, K76.7 |
| AIDS/HIV | 196.x-199.x | C77.x-C80.x |
| Cerebrovascular disease | 042.x-044.x | B20.x-B22.x, B24.x |

eTable 2a Baseline characteristics after IPTW

|  | N | % | Gefitinib | | Erlotinib | | Afatinib | | P value |
| --- | --- | --- | --- | --- | --- | --- | --- | --- | --- |
|  |  |  | N | % | N | % | N | % |  |
| **Total** | 10027 |  | 3683 |  | 3318 |  | 3026 |  |  |
| **Age** |  |  |  |  |  |  |  |  |  |
| Mean (±SD) | 67.4 | 11.8 | 67.4 | 12.4 | 67.4 | 11.8 | 67.4 | 11.3 | 0.990 |
| 20-64 | 4111 | 41.00 | 1518 | 41.22 | 1344 | 40.49 | 1250 | 41.29 | 0.764 |
| ≧65 | 5916 | 59.00 | 2165 | 58.78 | 1975 | 59.51 | 1777 | 58.71 |  |
| **Gender** |  |  |  |  |  |  |  |  |  |
| Male | 3737 | 37.27 | 1370 | 37.19 | 1240 | 37.37 | 1127 | 37.24 | 0.987 |
| Female | 6290 | 62.73 | 2313 | 62.81 | 2078 | 62.63 | 1899 | 62.76 |  |
| **Diagnosed year** |  |  |  |  |  |  |  |  |  |
| 2014 | 2345 | 23.38 | 913 | 24.80 | 861 | 25.93 | 571 | 18.86 | <.001 |
| 2015 | 2497 | 24.90 | 896 | 24.33 | 717 | 21.59 | 884 | 29.23 |  |
| 2016 | 2489 | 24.82 | 866 | 23.52 | 798 | 24.04 | 825 | 27.26 |  |
| 2017 | 2697 | 26.89 | 1007 | 27.35 | 943 | 28.43 | 746 | 24.65 |  |
| **Comorbidity** |  |  |  |  |  |  |  |  |  |
| Myocardial infarction | 89 | 0.88 | 33 | 0.88 | 30 | 0.91 | 26 | 0.85 | 0.968 |
| Congestive heart failure | 585 | 5.83 | 218 | 5.93 | 194 | 5.84 | 173 | 5.72 | 0.933 |
| Peripheral vascular disease | 146 | 1.45 | 54 | 1.48 | 46 | 1.37 | 46 | 1.51 | 0.894 |
| Cerebrovascular disease | 1056 | 10.53 | 381 | 10.36 | 349 | 10.52 | 325 | 10.75 | 0.874 |
| Dementia | 333 | 3.32 | 123 | 3.34 | 112 | 3.37 | 98 | 3.25 | 0.961 |
| Chronic pulmonary disease | 2778 | 27.71 | 1034 | 28.09 | 920 | 27.72 | 824 | 27.23 | 0.739 |
| Rheumatic disease | 120 | 1.19 | 45 | 1.22 | 40 | 1.19 | 35 | 1.16 | 0.971 |
| Peptic ulcer disease | 1451 | 14.47 | 540 | 14.66 | 484 | 14.59 | 427 | 14.11 | 0.792 |
| Mild liver disease | 631 | 6.29 | 235 | 6.39 | 211 | 6.36 | 185 | 6.10 | 0.874 |
| Diabetes without chronic complication | 2046 | 20.40 | 748 | 20.32 | 680 | 20.50 | 617 | 20.40 | 0.983 |
| Diabetes with chronic complication | 535 | 5.33 | 198 | 5.38 | 177 | 5.34 | 159 | 5.27 | 0.978 |
| Hemiplegia/paraplegia | 99 | 0.99 | 38 | 1.02 | 33 | 1.00 | 28 | 0.93 | 0.922 |
| Renal disease | 588 | 5.87 | 218 | 5.92 | 196 | 5.92 | 174 | 5.75 | 0.945 |
| Moderate-severe liver disease | 6 | 0.06 | <5 |  | <5 |  | <5 |  |  |
| Metastatic solid tumor | 6325 | 63.08 | 2313 | 62.80 | 2097 | 63.18 | 1916 | 63.31 | 0.902 |
| AIDS/HIV | <5 |  | <5 |  | <5 |  | <5 |  |  |

eTable 2a Baseline characteristics after IPTW (continue)

|  | N | % | Gefitinib | | Erlotinib | | Afatinib | | P value |
| --- | --- | --- | --- | --- | --- | --- | --- | --- | --- |
|  |  |  | N | % | N | % | N | % |  |
| **CCI score** |  |  |  |  |  |  |  |  |  |
| 0 | 1303 | 12.99 | 492 | 13.35 | 417 | 12.57 | 394 | 13.01 | 0.889 |
| 1 | 1224 | 12.21 | 453 | 12.30 | 401 | 12.07 | 370 | 12.23 |  |
| ≧2 | 7500 | 74.80 | 2738 | 74.34 | 2500 | 75.35 | 2262 | 74.75 |  |
| **Brain metastases** |  |  |  |  |  |  |  |  |  |
| Yes | 1987 | 19.81 | 717 | 19.46 | 661 | 19.91 | 610 | 20.14 | 0.771 |
| No | 8040 | 80.19 | 2966 | 80.54 | 2658 | 80.09 | 2417 | 79.86 |  |

IPTW: inverse probability of treatment weighting; SD: standard deviation; AIDS: acquired immunodeficiency syndrome; HIV: Human Immunodeficiency Virus; CCI: Charlson Comorbidity Index

eTable 2b Baseline characteristics after IPTW and propensity score matching

|  | N | % | Gefitinib | | Erlotinib | | Afatinib | | P value | Gefitinib v.s. Erlotinib | Gefitinib v.s. Afatinib | Erlotinib v.s. Afatinib |
| --- | --- | --- | --- | --- | --- | --- | --- | --- | --- | --- | --- | --- |
|  |  |  | N | % | N | % | N | % |  | SMD | SMD | SMD |
| **Total** | 6675 |  | 2225 |  | 2225 |  | 2225 |  |  |  |  |  |
| **Age** |  |  |  |  |  |  |  |  |  |  |  |  |
| Mean (±SD) | 67.26 | 11.3 | 67.04 | 11.7 | 67.44 | 11.6 | 67.30 | 10.5 | 0.486 | 0.03 | 0.02 | 0.01 |
| 20-64 | 2743 | 41.09 | 925 | 41.57 | 897 | 40.31 | 921 | 41.39 | 0.653 | 0.03 | 0.00 | 0.02 |
| ≧65 | 3932 | 58.91 | 1300 | 58.43 | 1328 | 59.69 | 1304 | 58.61 |  |  |  |  |
| **Gender** |  |  |  |  |  |  |  |  |  |  |  |  |
| Male | 2388 | 35.78 | 784 | 35.24 | 828 | 37.21 | 776 | 34.88 | 0.216 | 0.04 | 0.01 | 0.05 |
| Female | 4287 | 64.22 | 1441 | 64.76 | 1397 | 62.79 | 1449 | 65.12 |  |  |  |  |
| **Diagnosed year** |  |  |  |  |  |  |  |  |  |  |  |  |
| 2014 | 1033 | 15.48 | 406 | 18.25 | 316 | 14.20 | 311 | 13.98 | <.001 | 0.11 | 0.12 | 0.01 |
| 2015 | 1832 | 27.45 | 575 | 25.84 | 613 | 27.55 | 644 | 28.94 |  | 0.04 | 0.07 | 0.03 |
| 2016 | 1934 | 28.97 | 601 | 27.01 | 661 | 29.71 | 672 | 30.20 |  | 0.06 | 0.07 | 0.01 |
| 2017 | 1876 | 28.10 | 643 | 28.90 | 635 | 28.54 | 598 | 26.88 |  | 0.01 | 0.05 | 0.04 |
| **Comorbidity** |  |  |  |  |  |  |  |  |  |  |  |  |
| Myocardial infarction | 57 | 0.85 | 16 | 0.72 | 21 | 0.94 | 20 | 0.90 | 0.690 | 0.02 | 0.02 | 0.00 |
| Congestive heart failure | 369 | 5.53 | 132 | 5.93 | 108 | 4.85 | 129 | 5.80 | 0.230 | 0.05 | 0.01 | 0.04 |
| Peripheral vascular disease | 94 | 1.41 | 29 | 1.30 | 36 | 1.62 | 29 | 1.30 | 0.589 | 0.03 | 0.00 | 0.03 |
| Cerebrovascular disease | 620 | 9.29 | 195 | 8.76 | 229 | 10.29 | 196 | 8.81 | 0.136 | 0.05 | 0.00 | 0.05 |
| Dementia | 210 | 3.15 | 74 | 3.33 | 75 | 3.37 | 61 | 2.74 | 0.407 | 0.00 | 0.03 | 0.04 |
| Chronic pulmonary disease | 1899 | 28.45 | 600 | 26.97 | 646 | 29.03 | 653 | 29.35 | 0.160 | 0.05 | 0.05 | 0.01 |
| Rheumatic disease | 80 | 1.20 | 27 | 1.21 | 27 | 1.21 | 26 | 1.17 | 0.987 | 0.00 | 0.00 | 0.00 |
| Peptic ulcer disease | 933 | 13.98 | 325 | 14.61 | 296 | 13.30 | 312 | 14.02 | 0.454 | 0.04 | 0.02 | 0.02 |
| Mild liver disease | 431 | 6.46% | 142 | 6.38% | 145 | 6.52% | 144 | 6.47% | 0.983 | 0.01 | 0.00 | 0.00 |

| Diabetes without chronic complication | 1384 | 20.73 | 457 | 20.54 | 490 | 22.02 | 437 | 19.64 | 0.141 | 0.04 | 0.02 | 0.06 |
| --- | --- | --- | --- | --- | --- | --- | --- | --- | --- | --- | --- | --- |
| Diabetes with chronic complication | 348 | 5.21 | 111 | 4.99 | 133 | 5.98 | 104 | 4.67 | 0.125 | 0.04 | 0.01 | 0.06 |
| Hemiplegia/paraplegia | 59 | 0.88 | 22 | 0.99 | 23 | 1.03 | 14 | 0.63 | 0.287 | 0.00 | 0.04 | 0.04 |
| Renal disease | 337 | 5.05 | 102 | 4.58 | 118 | 5.30 | 117 | 5.26 | 0.471 | 0.03 | 0.03 | 0.00 |
| Moderate-severe liver disease | 6 | 0.09 | <5 |  | <5 |  | <5 |  |  | 0.02 | 0.01 | 0.03 |
| Metastatic solid tumor | 3997 | 59.88% | 1316 | 59.15% | 1350 | 60.67% | 1331 | 59.82% | 0.581 | 0.03 | 0.01 | 0.02 |
| AIDS/HIV | <5 |  | <5 |  | <5 |  | <5 |  |  | 0.00 | 0.03 | 0.03 |
| **CCI score** |  |  |  |  |  |  |  |  |  |  |  |  |
| 0 | 1303 | 12.99 | 492 | 13.35 | 417 | 12.57 | 394 | 13.01 | 0.889 | 0.03 | 0.01 | 0.02 |
| 1 | 1224 | 12.21 | 453 | 12.30 | 401 | 12.07 | 370 | 12.23 |  | 0.03 | 0.01 | 0.03 |
| ≧2 | 7500 | 74.80 | 2738 | 74.34 | 2500 | 75.35 | 2262 | 74.75 |  | 0.04 | 0.00 | 0.04 |
| **Brain metastases** |  |  |  |  |  |  |  |  |  |  |  |  |
| Yes | 1987 | 19.81 | 717 | 19.46 | 661 | 19.91 | 610 | 20.14 | 0.771 | 0.01 | 0.01 | 0.00 |
| No | 8040 | 80.19 | 2966 | 80.54 | 2658 | 80.09 | 2417 | 79.86 |  |  |  |  |

eTable 2b Baseline characteristics after IPTW and propensity score matching (continue)

IPTW: inverse probability of treatment weighting; SD: standard deviation; SMD: standard mean difference; AIDS: acquired immunodeficiency syndrome; HIV: Human Immunodeficiency Virus; CCI: Charlson Comorbidity Index

eTable 3 Cox proportional hazard model for overall survival after propensity score matching

|  | Univariate analysis | | | Multivariable analysis | | |
| --- | --- | --- | --- | --- | --- | --- |
| Variables | Crude HR | 95% CI | P value | Adjusted HR | 95% CI | P value |
| **EGFR-TKI** |  |  |  |  |  |  |
| Erlotinib vs Gefitinib | 1.018 | 0.911-1.137 | 0.756 | 0.941 | 0.846-1.047 | 0.263 |
| Afatinib vs Gefitinib | 0.787 | 0.702-0.883 | <.001 | 0.819 | 0.735-0.912 | <.001 |
| Afatinib vs Erlotinib | 0.883 | 0.790-0.987 | 0.028 | 0.870 | 0.781-0.968 | 0.011 |
| **Age** |  |  |  |  |  |  |
| 20-64 | 1.000 |  |  | 1.000 |  |  |
| ≧65 | 1.585 | 1.400-1.795 | <.001 | 2.605 | 2.003-3.388 | <.001 |
| **Gender** |  |  |  |  |  |  |
| Male | 1.000 |  |  | 1.000 |  |  |
| Female | 0.561 | 0.437-0.719 | <.001 | 0.606 | 0.468-0.786 | <.001 |
| **CCI score** |  |  |  |  |  |  |
| 0 | 1.000 |  |  | 1.000 |  |  |
| 1 | 1.196 | 0.956-1.496 | 0.118 | 1.159 | 0.922-1.456 | 0.206 |
| ≧2 | 1.456 | 1.170-1.813 | <.001 | 1.309 | 1.043-1.644 | 0.020 |
| **Brain metastases** |  |  |  |  |  |  |
| No | 1.000 |  |  | 1.000 |  |  |
| Yes | 1.584 | 1.175-2.135 | 0.003 | 1.688 | 1.227-2.322 | 0.001 |

HR: hazard ratio; CI: confidence interval; EGFR-TKI: epidermal growth factor receptor- tyrosine kinase inhibitor; CCI: Charlson Comorbidity Index

eTable 4 Cox proportional hazard model for time to treatment failure after propensity score matching

|  | Univariate analysis | | | Multivariable analysis | | |
| --- | --- | --- | --- | --- | --- | --- |
| Variables | Crude HR | 95% CI | P value | Adjusted HR | 95% CI | P value |
| **EGFR-TKI** |  |  |  |  |  |  |
| Erlotinib vs Gefitinib | 1.020 | 0.928-1.120 | 0.683 | 0.974 | 0.892-1.064 | 0.560 |
| Afatinib vs Gefitinib | 0.771 | 0.701-0.848 | <.001 | 0.869 | 0.764-0.988 | 0.032 |
| Afatinib vs Erlotinib | 0.818 | 0.745-0.899 | <.001 | 0.892 | 0.784-1.015 | 0.083 |
| **Age** |  |  |  |  |  |  |
| 20-64 | 1.000 |  |  | 1.000 |  |  |
| ≧65 | 1.142 | 1.030-1.265 | 0.012 | 1.469 | 1.248-1.728 | <.001 |
| **Gender** |  |  |  |  |  |  |
| Male | 1.000 |  |  | 1.000 |  |  |
| Female | 0.695 | 0.566-0.852 | <.001 | 0.688 | 0.557-0.849 | <.001 |
| **CCI score** |  |  |  |  |  |  |
| 0 | 1.000 |  |  | 1.000 |  |  |
| 1 | 1.071 | 0.899-1.276 | 0.443 | 1.044 | 0.875-1.247 | 0.631 |
| ≧2 | 1.168 | 0.986-1.384 | 0.072 | 1.086 | 0.912-1.295 | 0.353 |
| **Brain metastases** |  |  |  |  |  |  |
| No | 1.000 |  |  | 1.000 |  |  |
| Yes | 1.411 | 1.103-1.806 | 0.006 | 1.505 | 1.163-1.948 | 0.002 |

HR: hazard ratio; CI: confidence interval; EGFR-TKI: epidermal growth factor receptor- tyrosine kinase inhibitor; CCI: Charlson Comorbidity Index
